# The management of *Helicobacter pylori* infection and prevention and control of gastric cancer in China

**DOI:** 10.3389/fcimb.2022.1049279

**Published:** 2022-12-01

**Authors:** Yi Hu, Yin Zhu, Nong-Hua Lu

**Affiliations:** ^1^ Department Of Gastroenterology, Digestive Disease Hospital, The First Affiliated Hospital of Nanchang University, Nanchang, Jiangxi, China; ^2^ JiangXi Clinical Research Center for Gastroenterology, Nanchang, Jiangxi, China

**Keywords:** *Helicobacter pylori*, prevalence, resistance, eradication, gastric cancer

## Abstract

*Helicobacter pylori* (*H. pylori*) infection, a type-1 carcinogen, was closely associated with gastric cancer (GC). Successfully eradicating *H. pylori* infection could reduce the incidence of GC. China was a country with high incidence of GC and high prevalence of *H. pylori* infection. Nearly half of worldwide GC new cases and deaths attributed to *H. pylori* infection occurred in China. *H. pylori* prevalence varied over time with the improvement of socioeconomic status and sanitary conditions. The knowledge of antibiotic resistance rate in time was important to guide the clinical choice of antibiotics use in the regimens. With the publication of five Chinese consensus reports on the management of *H. pylori* infection and the effort of public preach of *H. pylori*-related knowledge, the standardization of *H. pylori* diagnosis and treatment by clinicians was improved. Bismuth-containing quadruple therapy was widely applied in clinical practice of *H. pylori* eradication because of high efficacy and safety. High-dose Proton Pump Inhibitor-amoxicillin dual therapy or vonoprazan-amoxicillin dual therapy showed comparable efficacy and lower side effects than bismuth-containing quadruple therapy, which were the alternative choice. The diagnosis rate of early GC was low and distinguishing Chinese GC risk population for the further endoscopy screening was important. Efforts have been done to establish prediction models to stratify GC risk in the Chinese GC risk population. We reviewed the current situation of the management of *H. pylori* infection and prevention and control of GC in China here.

## Introduction

In China, lung, colorectum and gastric cancer (GC) was the most common diagnosed cancer (Xia et al., 2022). The new case number and death number attributed to GC in China was 456, 124 and 390, 182 in 2018 (Bray et al., 2018). Meanwhile, the prevalence of *Helicobacter pylori* (*H. pylori*) infection was high in China, especially in area with high incidence of GC (Xie and Lu, 2015; Hooi et al., 2017). *H. pylori* infection was the main risk factor of GC (Malfertheiner et al., 2017), which was defined as type-1 carcinogen by the World Health Organization in 1994 and listed as the carcinogen by the 15th Report on carcinogens released by The U.S. Department of Health and Human Services in 2021. Nearly 90% of non-cardia GC were attributed to *H. pylori* infection, *H. pylori*-induced GC were estimated to be 810, 000 cases in 2018, which exceeded human papillomavirus-induced cervical cancer and hepatitis B virus-induced liver cancer, ranking the first among infection-attributable cancer (de Martel et al., 2020). China accounted for a third of worldwide cancer cases attributable to infection, especially *H. pylori* infection. We reviewed the current management of *H. pylori* infection and prevention and control of GC in China here.

## *Helicobacter pylori* prevalence

*H. pylori* was a common infection worldwide, with different prevalence among different regions. *H. pylori* was mainly acquired in childhood. *H. pylori* gastritis was defined as an infectious disease by *Kyoto global consensus report on Helicobacter pylori gastritis* (Sugano et al., 2015), whose transmission route included oral-oral, fecal-oral and gastro-oral (Leja et al., 2016). As such, *H. pylori* was closely related with socioeconomic status and sanitary conditions (Goh et al., 2011). In 1990, China was a country with low human development index (value: 0.499), which gradually increased to 0.761 (defined as high human development index) in 2018. Hooi et al. (2017) included 26 studies (103, 128 subjects) conducted in China from 1983-2013 for pooled analysis of *H. pylori* prevalence, the results demonstrated that the overall infection rate of *H. pylori* was 55.82%, which indicated that estimated more than 700 million subjects in China were infected with *H. pylori*. However, the interval of included studies were 20 years, which might not reflect the current situation of *H. pylori* prevalence. Moreover, the studies published in Chinese might be missed in this analysis. A slightly decreased *H. pylori* infection rate was observed around the world (2000-2008: 46.8% vs. 2009-2016: 44.9%) (Zamani et al., 2018). Consistent with this trend, the prevalence of *H. pylori* infection was decreased over time in China from 1983 to 2013 (Nagy et al., 2016). The decreased prevalence of *H. pylori* infection was more obvious in urban areas in comparison with rural areas. The overall *H. pylori* prevalence was higher in rural areas (66%) than urban areas (47%), which might be attributed to the differences of socioeconomic status and sanitary conditions. The weighted mean prevalence of *H. pylori* infection increased from 48% among individuals aged 18-30 years to 59% among individuals aged 50-60 years.

A recent systematic review and meta-analysis focusing on *H. pylori* prevalence in China included 412 eligible studies (published in English or Chinese) with 1, 377, 349 subjects for pooled analysis of *H. pylori* prevalence (Ren et al., 2022). *H. pylori* infection rate was reported as 44.2% (range 35.8%-66.4%) in the mainland of China in this study, which indicated that estimated 589 million individuals were *H. pylori* positive. *H. pylori* prevalence were high (>60%) in developing regions (Xizang, Guizhou etc.) and low (<40%) in developed regions (Beijing, Chongqing, Tianjin etc.). The significantly decreased *H. pylori* prevalence was also observed (1983-1994: 58.3% vs. 2015-2019: 40%). The infection rate of *H. pylori* was 28.0% in children and adolescents and 46.1% in adults. The overall global infection rate of *H. pylori* in children was 32.3%. *H. pylori* prevalence was higher in older children (41.6% in 13-18 years old) than in younger children (33.9% in 7-12 years old and 26.0% in 0-6 years old). Lower economic status, more siblings or children, room sharing, no access to a sewage system, having a mother or a sibling or siblings infected with *H. pylori*, drinking unboiled or non-treated water and older age were significantly associated with paediatric *H. pylori* infection (Yuan et al., 2022). Considering the improvement of socioeconomic status and sanitary conditions, it was not surprised that *H. pylori* prevalence had been decreased during the past decades. However, high infection rate of *H. pylori* was reported in developing areas or regions with high incidence of GC. Eradicating *H. pylori* effectively is important for decreasing *H. pylori* prevalence. Moreover, forming good health habits are effective for prevention of *H. pylori* transmission. Zeng et al. (2015) reported that the oral recombinant *H. pylori* vaccine was effective, safe, and immunogenic for *H. pylori*-naive children, which could substantially reduce the incidence of *H. pylori* infection. Further *H. pylori* vaccine studies are still needed to explore the protection of the vaccine against *H. pylori* infection and *H. pylori*-associated diseases during a long period.

## *Helicobacter pylori* recurrence

*H. pylori* recurrence remained a significant public health concern, which consisted of recrudescence or reinfection. Recrudescence was defined as reappearance of the original infection during short-term period (6 months), which was associated with the use of low efficient *H. pylori* regimen (Bell et al., 1993). Reinfection was defined as infection with a new strain of *H. pylori* occurred after 6 months, which required reexposure and was therefore more likely occurred in countries with high *H. pylori* prevalence and poor sanitation (Niv and Hazazi, 2008). We included 132 studies (53, 934 patient-years) for analyzing global *H. pylori* recurrence rate and explored the influence factors (Hu et al., 2017c). The global annual *H. pylori* recurrence, reinfection and recrudescence rate were 4.3%, 3.1% and 2.2%, respectively. Only three researches (Mitchell et al., 1998; Zhou et al., 2003; Zhou et al., 2017) had been conducted in China to explore the *H. pylori* recurrence rate until the year 2017. The pooled annual *H. pylori* recurrence rate was 2.1%, which was relatively low. The included subjects from the three studies were in developed regions (Beijing, Guangzhou, Jinan etc.), which might not reflect the total *H. pylori* recurrence rate in the mainland of China, especially in regions with low socioeconomic status and sanitary conditions. We further analyzed the associations between *H. pylori* recurrence rate and human development index (reflecting the socioeconomic status), *H. pylori* prevalence (reflecting sanitary conditions) and different periods. *H. pylori* recurrence rate was inversely related to the human development index and directly related to *H. pylori* prevalence, which might be explained by that subjects living in a country with low socioeconomic status and high *H. pylori* prevalence had more possibility of exposure of *H. pylori*. *H. pylori* recurrence rate remained relatively stable during three periods (1990s, 2000s and 2010s).

Xue et al. (2019) prospectively enrolled patients with successful eradication in developed regions (Beijing and Jinan) from 2013 to 2014 to conduct ^13^C-urea breath test at one year and 3 years after therapy. 743 patients finished the 1-year follow-up, 13 patients were *H. pylori*-positive, which indicated that the annual recurrence rate was 1.75%. 607 patients finished the 3-year follow-up, 28 patients recurred, which indicated that the 3-year recurrence rate was 4.61% and the annual recurrence rate was 1.5%. The invasive diagnoses or treatments, the level of income, and the hygiene standard of dining out place were the independent factors influencing *H. pylori* recurrence. A higher annual recurrence rate of *H. pylori* (4.75%) was reported in Jiangjin district, Chongqing (Zhou, 2020). Drinking and dining out were independent risk factors of *H. pylori* recurrence. Consistent with this study, Zhang et al. (2020a) also reported a high annual *H. pylori* recurrence rate (18.8%) among children. The follow-up of these two studies was short (1 year) and the enrolled patients were limited to one region. We conducted a large-scale multicenter, prospective open cohort, observational study to evaluate *H. pylori* recurrence rate during long-term follow-up (2012-2018, 7454.3 person-year) (Xie et al., 2020). 5, 193 subjects at 18 hospitals across 15 provinces were enrolled. The overall annual *H. pylori* recurrence, reinfection and recrudescence rate were 3.1%, 1.5% and 5.1%, respectively. Specific properties of ethnic groups, education level, family history or residence location was found to be at higher risk for *H. pylori* reinfection. *H. pylori* recurrence rate was relatively low in China, which could not be considered as the competing consideration to eradicate *H. pylori* and implement the “screen and treatment” strategy of *H. pylori*. Additionally, a recent systematic review and meta-analysis provided the sufficient evidences that whole family-based *H. pylori* treatment could increase eradication rate and reduce recurrence rate compared with single-infected patient treatment approach (Zhao et al., 2021). A family-based *H. pylori* prevention and eradication strategy would be an effective approach to prevent its intra-familial transmission (Ding et al., 2022).

## *Helicobacter pylori* resistance

Antibiotic resistance of *H. pylori* was the important factor influencing the efficacy of *H. pylori* regimen (Hu et al., 2017a). For example, the efficacy of clarithromycin-containing regimens was shown to below 80% when the clarithromycin resistance rates were higher than 20%, which was then classified as unacceptable as the first-line treatment for *H. pylori* eradication (Graham et al., 2007). Clarithromycin resistance is due to mutations in the 23S rRNA gene. The 23S ribosomal RNA A2143G, A2142G, and A2142C mutations accounted for 80–90% of clarithromycin resistance. Similarly, levofloxacin resistance is mostly due to mutations at positions 87, 88, 91, 97 of gene *gyrA*. The occurrence of point mutations in the *pbp 1A* gene was the most common mechanism leading to moderate or low-level amoxicillin resistance. Resistance to tetracycline is mainly caused by point mutations in *tet*-1 in 16S rRNA genes. Metronidazole resistance is highly complex although *rdxA* mutations are highly predictive of metronidazole resistance (Peter et al., 2022). Furazolidone resistance is associated with Mutations in the *H. pylori porD* and *oorD* genes (Hu et al., 2017a). *H. pylori* resistance to common antibiotics (clarithromycin, metronidazole and levofloxacin) used in the regimen was quite serious (resistance rate >15%) in majority of World Health Organization Regions (Savoldi et al., 2018). In 2017, clarithromycin-resistant *H. pylori* was listed as antibiotic-resistant bacteria needing a high priority for antibiotic research and development of new antibiotics by World Health Organization (Tacconelli et al., 2018). In the Asia-Pacific region, the mean prevalence of primary *H. pylori* resistance was reported as 17% for clarithromycin, 44% for metronidazole, 18% for levofloxacin, 3% for amoxicillin, and 4% for tetracycline (Kuo et al., 2017). The resistance rate of clarithromycin and levofloxacin increased over time mainly after the year 2000. Primary *H. pylori* resistance to metronidazole remained stable but was above the alarming level from 1990 to 2016. Amoxicillin and tetracycline were considered to be susceptible antibiotics in this region. We also included 23 studies (published in English or Chinese) conducted in China focusing on primary antibiotic resistance of *H. pylori* to evaluate the overall pooled antibiotic resistance rate (Hu et al., 2017b). The results showed that the prevalence of *H. pylori* primary resistance to clarithromycin (28.9%), metronidazole (63.8%), and levofloxacin (28.0%) was high and increased over time. However, the resistance rates to amoxicillin (3.1%), tetracycline (3.9%), and furazolidone (1.7%) were low and stable over time.

Considering the differences of antibiotic resistance existed among different regions, we conducted a multi-region prospective 7-year study (2010-2016) to explore the characteristics of *H. pylori* resistance in China (Liu et al., 2018). *H. pylori* were successfully cultured from 1, 117 patients in 13 provinces or cities. An E-test was used to determine the minimum inhibitory concentrations of amoxicillin, metronidazole, clarithromycin, levofloxacin and tetracycline. The Kirby-Bauer disc diffusion method was used to determine the inhibition zone for furazolidone. The resistance rate of metronidazole, clarithromycin and levofloxacin was above the alarming level (>15%), as well as the dual antibiotic resistance rate (metronidazole+clarithromycin or metronidazole+ levofloxacin). The resistance rate of amoxicillin, tetracycline and furazolidone resistance was relatively low (<4%). The resistance rate of antibiotics was higher in subjects with age ≥40 years than subjects<40 years old, which might be explained by the exposure time of antibiotics were longer in the elder subjects. Additionally, the resistance rate of metronidazole, clarithromycin and amoxicillin were significantly different among different regions of China. Recently, A retrospective cross-sectional observational study was conducted by the China Center for *H. pylori* Molecular Medicine to investigate resistance of *H. pylori* strains to antibiotics from 2018 to 2020 across different regions of China. The results included 4, 242 *H. pylori* strains. Kirby-Bauer disk diffusion method was used to determine the phenotypic resistance to antibiotics. Overall, the primary antibiotic resistance rates of *H. pylori* for clarithromycin, levofloxacin and metronidazole were above the alarming level (>15%). The primary antibiotic resistance rates of *H. pylori* for amoxicillin, furazolidone and tetracycline were relatively low (<4%) except in Northwest China (Zhong et al., 2021). It should be noted that the primary resistance rate of *H. pylori* for antibiotics in specific region of China (e.g., Northwest China) showed an increasing trend. The selection of antibiotics in the regimens should base on the antibiotic resistance rate in local area. The previously treated adult group exhibited higher resistance rate of antibiotics (99.2% for metronidazole, 58.3% for clarithromycin and 52.3% for levofloxacin) compared with treatment-naive adult (Liu et al., 2019). As such, elevating the efficacy of first-line treatment for *H. pylori* is important, which could avoid the occurrence of secondary resistance. The situation of *H. pylori* resistance was serious in China. The combinations of susceptible antibiotics (amoxicillin+furazolidone or amoxicillin+tetracycline or tetracycline+furazolidone) were recommended by the *Fifth Chinese National Consensus Report on the management of Helicobacter pylori infection* (Liu et al., 2018). Real-time monitoring of antibiotic resistance was also needed to further guide the selection of antibiotics used in the *H. pylori* regimens.

## National survey regarding the management of *Helicobacter pylori* infection

In 2013, *Fourth Chinese National Consensus Report on the management of Helicobacter pylori infection* was published, including the indications, diagnosis and treatment of *H. pylori*. Proton Pump Inhibitor (PPI)-based triple therapy was not recommended as the first-line treatment of *H. pylori* infection because of its low efficacy (<80%). Bismuth-containing quadruple therapy (BQT) was recommended as the first-line and second-line regimen for eradicating *H. pylori*, the duration of *H. pylori* regimens increased from 7 days to 10 or 14 days (Liu et al., 2013). In 2018, *Fifth Chinese National Consensus Report on the management of Helicobacter pylori infection* was published, which contained 6 aspects including *H. pylori* indications for eradication, diagnosis, treatment, *H. pylori* and GC, *H. pylori* infection in special populations, *H. pylori* and gastrointestinal microbiota (Liu et al., 2018). *H. pylori* gastritis is defined as an infectious disease, which was consistent with the recommendation reported in the *Kyoto global consensus report on Helicobacter pylori gastritis*. *H. pylori* infection confirmed was included in the *H. pylori* indications. Considering the serious situation of antibiotic resistance, BQT (recommended 7 regimens) was still recommended as the main empirical therapy for *H. pylori* eradication because of its high efficacy especially for *H. pylori* resistant strains. Compared to *Fourth Chinese National Consensus Report*, two regimens of BQT (PPI+bismuth+amoxicillin+metronidazole, PPI+bismuth+amoxicillin+tetracycline) were extended. In order to investigate the management of *H. pylori* infection by clinicians in China, the Chinese Study Group on *H. pylori* conducted an authoritative survey at 14th (2014) and 17th (2017) Congress of Gastroenterology China (Song et al., 2019b). A total of 4, 182 valid samples were obtained in this study. Majority of the clinicians were aware of *H. pylori*-related diseases (chronic gastritis, peptic ulcer, GC and gastric MALT lymphoma). However, less than 50% of clinicians were familiar with the associations between *H. pylori* infection and extra‐gastroduodenal diseases. Urea breath test were the preferred choice for detecting *H. pylori* infection by 80% of the clinicians. The demands for *H. pylori* detection by patients were increased mainly because of the increased awareness of *H. pylori* harmfulness by the publics and the preach of *H. pylori* consensus reports by the gastroenterologists. The proportion rate of quadruple therapy as the first-line treatment for *H. pylori* infection was higher (76%) in 2017 than that in 2014 (31%). The preferred antibiotic combinations were clarithromycin + amoxicillin, followed by amoxicillin + furazolidone. The combination of clarithromycin and metronidazole was used for the initial eradication by only 1% of the clinician because the dual resistance rate of clarithromycin and metronidazole was high (>15%). From this survey, we found that the management of *H. pylori* infection has been improved. However, the proportion rate of BQT was only 57% in 2017 and the frequency of resistant antibiotics (clarithromycin, metronidazole and levofloxacin) used in the regimens were still high.

After 4 years, the Chinese Study Group on *H. pylori* conducted a nationwide, multicenter, cross-sectional questionnaire survey again to investigate the current state of knowledge and practice of *H. pylori* infection management (Song et al., 2022). The proportion of BQT was high (88%) in the first-line treatment of *H. pylori* infection. Amoxicillin was the most preferred antibiotic (93.1%) used in the regimen. The preferred antibiotic combinations were still clarithromycin + amoxicillin, followed by amoxicillin+furazolidone. The proportion of regimens used for 14 days or 10 days were 83.3% and 13.5%, respectively. Compared to the survey in 2017, the treatment of *H. pylori* infection was more standard in 2021. The awareness of *H. pylori* prevention and treatment in the general Chinese population was also important, an internet-based survey was carried out in 2019 by the National Clinical Research Center for Digestive Diseases, which enrolled 3, 211 people and 546 physicians (Wu et al., 2020). The majority of the surveyed population (87.0%) and physicians (82.2%) supported a national *H. pylori* screening plan to prevent GC. However, the proportion of subjects who answered correctly to all questions about *H. pylori*’s infectivity, harmfulness and preventive measures were only 16%, 35% and 43.6%, respectively. More works on health education are needed because the general population in China has insufficient awareness of *H. pylori*.

## *Helicobacter pylori* treatment

### Bismuth-containing quadruple therapy

Standard PPI-based triple therapy was not recommended for *H. pylori* eradication because of its low efficiency. The monotherapy of bismuth could directly eradicate 20% *H. pylori* successfully. The addition of bismuth in triple therapy might improve the cure rate, mainly in strains with antibiotic resistance (Dore et al., 2016). Numerous studies have evaluated the efficacy of different antibiotic combinations in BQT in China, including clarithromycin and amoxicillin (Sun et al., 2010), amoxicillin and furazolidone (Xie et al., 2014), amoxicillin and metronidazole (Zhang et al., 2015), amoxicillin and tetracycline (Xie et al., 2018b), tetracycline and metronidazole (Liang et al., 2013), tetracycline and furazolidone (Liang et al., 2013). The efficacy of different antibiotic combinations in BQTs was good or fair in most studies, with the intention-to-treat (ITT) analysis showing an efficacy above 85% and the per protocol (PP) analysis showing an efficacy above 90%. As such, BQT was recommended as the first-line treatment of *H. pylori* infection by the *Fifth Chinese National Consensus Report on the management of Helicobacter pylori infection* (Liu et al., 2018). The duration of regimens should be 14 days, except evidences showed that regimens for 10 days achieved high efficacy. Susceptible antibiotics (amoxicillin, tetracycline and furazolidone) were the preferred choices in the regimens. We performed a national, multicenter, open-label, randomized controlled trial to evaluate the efficacy of the combination of susceptible antibiotic (amoxicillin and furazolidone) in BQT for eradicating *H. pylori* (Xie et al., 2018a). A high efficacy (94.7%) and low side effect (9.7%) were achieved by furazolidone-containing BQT for 10 days. Additionally, an observational study of furazolidone-containing BQT for *H. pylori* infection in real-world settings was conducted from 2015 to 2018, the eradication rate of furazolidone-containing BQT for 10 days or 14 days was 93.7% and 98.2%, respectively. Meanwhile, low side effect (<20%) was also observed for this regimen (Song et al., 2019a). Another combination of susceptible antibiotic (amoxicillin and tetracycline) in BQT for 10 days was showed to achieve 91.9% eradication rate as the first-line treatment for *H. pylori* infection, which was confirmed by multicenter, randomized, parallel-controlled clinical trial (Xie et al., 2018b).

Public attentions were paid to the alterations of gastric or intestinal microbiota induced by BQT because antibiotics and PPI used in the regimes might influence the host microbiota to some extent. We included ten asymptomatic young adults with *H pylori*-related gastritis treated with BQT for 14 days, 7 age-matched adults with *H. pylori* negative were served as healthy controls. Both fecal and gastric mucosa samples were collected for 16S rRNA gene sequencing at the timepoint of before eradication, after eradication and 6 months after the therapy. The results demonstrated that BQT can restore the diversity of gastric microbiota with enrichment of beneficial bacteria. The composition of gut microbiota after *H. pylori* eradication trends toward healthy status (He et al., 2019). The main drawback of BQT was the complex of drug administration, which might influence the compliance of the subjects, leading to the decreased efficacy of regimen. Enhanced educational interventions were effective way to improve the adherence among infected patients (Zha et al., 2022). WeChat is a social media platform widely used in China. Luo et al. (2020) found that *H. pylori*-infected subjects intervened with WeChat as a reminder tool during the treatment exhibited better disease-related knowledge, medication adherence, and eradication rates than that in the control group. The following research with more *H. pylori*-infected subjects enrolled further confirmed this finding (Ma et al., 2021). Compared to the conventional patient education (verbal education and a specifically designed printout with detailed instructions), the addition of WeChat-based patient-doctor interaction did not yield better results of efficacy or compliance (Lin et al., 2022). Selecting efficient antibiotic combinations in BQT should depend on the local *H. pylori* resistance. Enhanced patient education (conventional education or social media platform interactions) was also important for the success of *H. pylori* regimen.

### High-dose PPI-amoxicillin dual therapy or vonoprazan-amoxicillin dual therapy

The role of PPI in *H. pylori* regimen was to maintain the gastric pH high enough for *H. pylori* to remain in the state of active replication where the organism becomes susceptible to amoxicillin (Graham and Fischbach, 2010) The eradication rate of *H. pylori* increased when the PPI dosage used in the regimen increased (Labenz, 2001). High-dose dual therapy (HDDT) was defined as the combination of amoxicillin and PPI in high dose for 14 days, which was evaluated for *H. pylori* eradication in recent years in China. Yang et al. (2019) firstly evaluated HDDT (amoxicillin 750mg q.i.d and esomeprazole 20mg q.i.d) as the first-line treatment of *H. pylori.* 91.1% eradication rate was achieved by HDDT in adherent infected subjects, which was non-inferior to BQT. The study conducted in Beijing with same regimen also reported the similar high efficacy for HDDT (Song et al., 2020). Changing the frequency of amoxicillin to 1000mg t.i.d in HDDT was also reported to achieve high efficacy (ITT: 89.4%, PP: 90.6%) (Guan et al., 2022). Increasing the dosage of esomeprazole to 40 mg t.i.d in HDDT was shown to achieve 91.7% eradication rate for ITT analysis and 95.7% in PP analysis (Tai et al., 2019). The addition of bismuth in HDDT only improved treatment efficacy among smoker (Yu et al., 2019). The eradication rate of HDDT was low when the duration was 10 days or the esomeprazole was given 20 mg t.i.d (Zhang et al., 2020b). The combination of amoxicillin and rabeprazole (10mg t.i.d) or Ilaprazole (5 mg bid) for 14 days was reported to be with high efficiency for eradicating *H. pylori* (Niu et al., 2022; Shao et al., 2022). Additionally, the combination of amoxicillin (1000mg t.i.d) and rabeprazole (10mg t.i.d) were also efficient for *H. pylori* eradication in elder infected subjects or those with multiple comorbidities (Gao et al., 2020). In China, the duration of HDDT should be 14 days. The optimized dosage of amoxicillin was 1000mg t.i.d or 750 mg q.i.d, the dosage of PPIs should be double (i.e. esomeprazole 20mg q.i.d or 40 mg b.i.d) to maintain the high gastric pH level. HDDT showed non-inferior efficacy and lowed side effects rate than BQT in adults or elder patients (**Table 1**), which was considered as an alternative first-line treatment for *H. pylori* infection in China. However, the influence of HDDT on the gut microbiota remained unclear and need to be explored in the future research.

**Table 1:** Summary of HDDT or VA dual therapy as the first-line treatment of *H. pylori* infection in China.

Reference	Antibiotics	PPI or P-CAB	Days	n	ITT	PP	Adverse
Yang et al. (2019)	amoxicillin 750mg q.i.d	esomeprazole 20mg q.i.d	14	116	87.90%	91.10%	6.30%
Song et al. (2020)	amoxicillin 750mg q.i.d	esomeprazole 20mg q.i.d	14	380	87.10%	92.40%	17.60%
Guan et al. (2022)	amoxicillin 1000mg t.i.d	esomeprazole 20mg q.i.d	14	350	89.40%	90.60%	12.90%
Tai et al. (2019)	amoxicillin 750mg q.i.d	esomeprazole 40mg t.i.d	14	120	91.70%	95.70%	9.60%
Yu et al. (2019)	amoxicillin 1000mg t.i.d	esomeprazole 40mg b.i.d	14	80	92.50%	96.10%	7.50%
Zhang et al. (2020b)	amoxicillin 1000mg t.i.d	esomeprazole 20mg t.i.d	14	104	83.50%	86.40%	5.00%
	amoxicillin 750mg q.i.d	esomeprazole 20mg q.i.d	10	104	79.80%	81.30%	5.90%
Shao et al. (2022)	amoxicillin 1000mg t.i.d	rabeprazole 10mg t.i.d	14	120	85.80%	89.60%	13.00%
Niu et al. (2022)	amoxicillin 750mg q.i.d	Ilaprazole 5 mg bid	14	150	88.00%	93.00%	8.50%
Gao et al. (2020)	amoxicillin 1000mg t.i.d	rabeprazole 10mg t.i.d	14	198	–	90.90%	11.10%
Hu et al. (2022)	amoxicillin 1000mg b.i.d	vonoprazan 20mg b.i.d	10	37	89.20%	89.20%	29.70%
	amoxicillin 1000mg t.i.d	vonoprazan 20mg b.i.d	10	37	81.10%	81.10%	24.30%
	amoxicillin 1000mg b.i.d	vonoprazan 20mg b.i.d	7	24	66.70%	72.70%	16.70%
	amoxicillin 1000mg t.i.d	vonoprazan 20mg b.i.d	7	21	81.00%	81.00%	23.80%
Lin et al. (2022)	amoxicillin 750mg q.i.d	vonoprazan 20mg b.i.d	7	85	63.50%	65.10%	16.90%
	amoxicillin 500mg q.i.d	vonoprazan 20mg b.i.d	7	84	58.30%	66.20%	13.20%

PPI, proton‐pump inhibitor; P-CAB, potassium-competitive acid blocker; N, number; ITT, intention-to-treat; PP, per protocol; HDDT, High-dose dual therapy; VA, vonoprazan and amoxicillin. b.i.d, two times daily; t.i.d, three times daily; q.i.d, four times daily.

Vonoprazan, a potassium-competitive acid blocker, was firstly introduced in Japan, which inhibited the gastric acid faster, longer and stronger in comparison with PPIs (Abadi and Ierardi, 2019). Its combination with amoxicillin (750mg b.i.d or 500mg t.i.d) for 7 days in adults (Furuta et al., 2020); Suzuki et al., 2020) or junior high school students (Gotoda et al., 2020) achieved acceptable eradication rate in Japan. We have conducted a systematic review and meta-analysis to evaluate the efficacy and safety of vonoprazan and amoxicillin (VA) dual therapy (Ouyang et al., 2022), the results showed that the crude eradication rate of VA dual therapy was 87.5% and 89.6% by ITT and PP analysis, respectively. Additionally, the side effect of VA dual therapy was 19.1%. Japan government restrictions limit the duration of regimen to 7 days. Chey et al. (2022) further explored the efficacy of VA dual therapy (amoxicillin 1g t.i.d and vonoprazan 20 mg b.i.d) for 14 days in the United States and Europe. 349 *H. pylori*-infected subjects received VA dual therapy. The eradication rate of VA dual therapy was 77.2% in all patients. The efficacy of regimen was unsatisfied although it showed superior to lansoprazole triple therapy. The different outcomes were achieved in Japan, United States and Europe, which might be explained by the differences of race and Body Mass Index. We conducted a prospective, randomized clinical pilot study to evaluate the efficacy of VA dual therapy in China (Hu et al., 2022b). 119 *H. pylori*-infected subjects were randomized to receive either low- or high-dose VA consisting of amoxicillin 1 g either b.i.d. or t.i.d plus VPZ 20 mg b.i.d for 7 or 10 days. Neither 7- or 10-day VA dual therapy with b.i.d. or t.i.d. amoxicillin provides satisfied efficacy (<90%). The following muti-centers study conducted in Lanzhou also showed that the efficacy of VA dual therapy for 7days was relatively low (Lin et al., 2022), which could be explained by the short duration of VA dual therapy and high antibiotic resistance of amoxicillin in this region (Zhong et al., 2021). VA dual therapy (VPZ 20mg bid with amoxicillin 1000mg b.i.d or t.i.d) for 14 days was shown to achieve high efficacy (eradication rate > 90%) as the first-line treatment for *H. pylori* infection in our center (unpublished data). For *H. pylori*-infected patients with history of treatment failure, the eradication rate of VA dual therapy (VPZ 20mg q.d or b.i.d with amoxicillin 1000mg t.i.d) for 14 days was 92.5%, which was effective and safe as rescue therapy for *H. pylori* infection (Gao et al., 2022). More importantly, our results showed that VA dual therapy showed minimal influence on the gut microbiota and short-chain fatty acids (Hu et al., 2022a). VA dual therapy for 14 days showed high efficacy and good safety for eradicating *H. pylori*. The included samples were limited and the application of VA dual therapy in different regions should be optimized. VA dual therapy is a promising *H. pylori* regimen in an era of increasing antibiotic resistance although more evidences are needed. Further prospective, multi-center, randomized control trail with more subjects included was needed to evaluate the efficacy and safety of low or high-dose VA dual therapy for 14 days in different regions of China.

## The benefit of *Helicobacter pylori* eradication in the prevention of GC

China was a country with a high risk of GC (>20 per 100 000 person-years), especially in some regions (Changle, Linqu, Wuwei, Yangzhong, Zhuanghe etc.) (Liou et al., 2020). 2, 258 participants with *H. pylori* positive in Linqu county, Shandong province were randomized into *H. pylori* eradication, vitamin supplementation, garlic supplementation, or their placebos, who were followed up for 22 years (1995-2017). The results demonstrated that *H. pylori* treatment significantly reduce reduced risk of death due to GC (odds ratio 0.48, 95% confidence interval 0.32 to 0.71) (Li et al., 2019). In Changle, Fujian province, 1, 630 asymptomatic, *H. pylori*-infected subjects were randomly assigned to receive *H. pylori* eradication or placebo, who were then follow-up for 26.5 years (1994-2020). Subjects receiving *H. pylori* eradication had a lower incidence of GC than their placebo counterparts (hazard ratio, 0.57; 95% CI, 0.33-0.98), the benefit was more obvious in subjects without premalignant gastric lesions or dyspepsia symptoms at baseline (Yan et al., 2022). Mass eradication of *H. pylori* infection was launched in 2004 and continued until 2018 for a high-risk Taiwanese population aged 30 years or older dwelling on Matsu Islands with *H. pylori* infection. The effectiveness of *H. pylori* eradication in reducing GC incidence was 53% (95% CI 30% to 69%). Additionally, the decreased presence and severity of atrophic gastritis and intestinal metaplasia was also observed (Chiang et al., 2021). Acceptable cure rate of *H. pylori* and low rate of adverse effects were achieved during mass screening and eradication of *H. pylori* (Lei et al., 2022).

The benefit of *H. pylori* eradication in the prevention of GC was confirmed by randomized control trails with large subjects included and long-term follow-up in China, which supported the mass screening and eradication of *H. pylori*. The benefit was more obvious in subjects without the incidence of precancerous lesions. The subjects with precancerous lesions also benefit from *H. pylori* eradication because the successful treatment could prevent the progression of diseases. For example, *H. pylori* eradication could reduce the incidence of metachronous GC in subjects with early GC undergoing endoscopic mucosal resection (relative risk= 0.49; 95% CI 0.34 to 0.70) (Ford et al., 2020). Additionally, in an analysis of data from a public hospital database with 73, 237 *H. pylori* positive subjects included on Hong Kong, *H. pylori* infection eradication reduced the risk of GC in subjects 60 years or older compared to the matched general population (Leung et al., 2018). As such, the Chinese consensus (Du et al., 2020; Ding et al., 2022) have emphasized the importance of early detection, diagnosis, and treatment of *H. pylori* to reduce the occurrence of GC and a family-based *H. pylori* prevention and eradication strategy to prevent its intra-familial transmission and related diseases. Additionally, national, provincial and regional specialist clinic of *H. pylori* infection was established by the office of *H. pylori* infection and GC prevention and control, which could promote the standard of *H. pylori* diagnosis and treatment.

## The stratification of risk for GC in the Chinese GC risk population

The 5-year survival rate was below 30% when advanced GC was diagnosed (Ajani et al., 2013). Early GC showed good prognosis (5-year survival rate >90%), which could be cured by the endoscopic submucosal dissection (Isobe et al., 2011). However, the diagnosis rate of early GC was low (<10%) in China. An effective way to improve the current serious situation of diagnosis and treatment of GC was the endoscopy screening and treatment in patients with high-risk of GC. The current national screening guideline for GC in China recommended screening beginning at age 40 years for all in the high-risk population (those residing in high-incidence areas for more than 3 years or who have *H. pylori* infection, a positive family history of GC, or risk factors for GC etc.). Estimated exceed 300 million individuals were defined as high-risk of GC and needed to receive the screening gastroscopy, which is not likely feasible in the clinical practice. An applicable risk prediction rule to further stratify risk for GC in the Chinese GC risk population was needed.

Tu et al. (2017) conducted a cross-sectional study for identifying high-risk individuals and predicting risk of developing GC in Zhuanghe county, a rural county of northern China with high incidence and mortality of GC. The screening program included individuals with 35–70 years old or upper gastrointestinal symptoms or a positive family history of GC. Totally, 9, 002 participants underwent gastroscopy were recruited by the end of 2012. The five biomarkers (especially PGII, the PGI/II ratio, and *H. pylori* sero-positivity) (Area Under the Curve=0.803) were identified to be associated with the presence of precancerous gastric lesions or GC at enrollment. From 2015 to 2017, The Gastrointestinal Early Cancer Prevention & Treatment Alliance of China conducted a nationwide multi-center cross-sectional study to identify individuals with a high risk prior to gastroscopy, which recruited 14, 929 individuals aged 40–80 years who went to hospitals for a GC screening gastroscopy (Cai et al., 2019). Seven variables (age, sex, PG I/II ratio, G-17 level, *H. pylori* infection, pickled food and fried food) were comprised as the novel GC risk prediction rule, with scores ranging from 0 to 25. 70.8% of total GC cases and 70.3% of early GC cases were detected when individuals with medium risk (score: 12-16) and high risk (score: 17-25) received gastroscopy. Endoscopy requirements could be reduced by 66.7% according to the low-risk proportion. This nationwide study successfully developed and validated prediction rule identifying individuals at a higher risk in a Chinese high-risk population, which needed to receive further gastroscopy. Compared to ABC method (Miki, 2011), five markers-based method (Tu et al., 2017), the prediction model of seven variables (Cai et al., 2019) showed higher Area Under the Curve and Youden index values, which was further confirmed by a retrospective analysis of data from the Provincial Gastric Cancer Screening Program with 97, 541 individuals included (Hu et al., 2021). A future large population-based screening project should be launched to test and validate its efficacy of novel GC risk prediction rule.

## Conclusions

China was a country with high incidence of GC and high infection rate of *H. pylori*. With the improvement of socioeconomic status and sanitary conditions and successful *H. pylori* eradication, the prevalence of *H. pylori* showed a decreased trend over time. Meanwhile, *H. pylori* recurrence rate remained low during the long-term follow-up. The situation of *H. pylori* resistant to antibiotics is serious worldwide, including in China. Suspectable antibiotics are recommended to be used in the regimens. With the publication of Chinese consensus report on the management of *H. pylori* infection and the effort of public preach of *H. pylori*-related knowledge, the standardization of diagnosis and treatment of *H. pylori* by clinicians was improved. Efforts were still needed to be done for education of public populations and the supervisor of *H. pylori* management in clinical practice. For example, the office for prevention and treatment of *H. pylori* infection and GC was established in 2021, which aimed to launch the national or provincial or county demonstration center of the standard outpatient clinic of *H. pylori* diagnosis and treatment. BQT was effective and safe, which was recommended as the first-line treatment for *H. pylori* infection. HDDT and VA dual therapy were the alternative choice. Successful *H. pylori* eradication reduce the incidence of GC and prevent the progression of *H. pylori*-related diseases. Prediction models had been developed and validated to stratify GC risk in the Chinese GC risk population.

“Healthy China 2030” proposed the improvement of public health with expectation of 5-year nationwide survival rate of cancer improved by 15%. The combination of primary (effective *H. pylori* eradication) and secondary prevention (increasing the diagnosis rate of early GC and treated them) was the effective way to decrease the incidence of GC and increase the survival rate of GC.

## Author contributions

YH wrote the manuscript. YZ and N-HL designed and revised the manuscript. All authors contributed to the article and approved the submitted version.

## Funding

This study was supported by the National Natural Science Foundation of China (No. 82000531 and 82170580), the Project for Academic and Technical Leaders of Major Disciplines in Jiangxi Province (No. 20212BCJL23065), the Key Research and Development Program of Jiangxi Province (No. 20212BBG73018), the Youth Project of the Jiangxi Natural Science Foundation (No. 20202BABL216006), National Science and Technology Award Reserve Cultivation Project (20192AEI91008) and The First Affiliated Hospital of Nanchang University Clinical Research and Cultivation Project (YFYLCYJPY202002).

## Conflict of interest

The authors declare that the research was conducted in the absence of any commercial or financial relationships that could be construed as a potential conflict of interest.

## Publisher’s note

All claims expressed in this article are solely those of the authors and do not necessarily represent those of their affiliated organizations, or those of the publisher, the editors and the reviewers. Any product that may be evaluated in this article, or claim that may be made by its manufacturer, is not guaranteed or endorsed by the publisher.
